# Simultaneous Quantification of Ten Active Components in Traditional Chinese Formula Sijunzi Decoction Using a UPLC-PDA Method

**DOI:** 10.1155/2014/570359

**Published:** 2014-05-20

**Authors:** Kang An, Guo Jin-rui, Zhang Zhen, Wang Xiao-long

**Affiliations:** ^1^School of Pharmacy, Nanjing University of Chinese Medicine, 138 Xianlin Road, Nanjing 210046, China; ^2^Discipline of Chinese and Western Integrative Medicine, Nanjing University of Chinese Medicine, 138 Xianlin Road, Nanjing 210046, China; ^3^State Key Laboratory of Natural Medicines, China Pharmaceutical University, Nanjing 210009, China

## Abstract

Sijunzi decoction (SJZT), a traditional Chinese formula (TCMF) consisting of four herbs, has been widely used for the treatment of various gastrointestinal symptoms. However, its modernization process is hindered by the lack of a powerful quality control method that covers the major active components in the formula. The aim of this study was to establish a UPLC method for the quantitative determination of ten active components in Sijunzi decoction including ginsenoside Rg_1_, Re, Rb_1_, liquiritin, liquiritigenin, glycyrrhizic acid, atractylenolide I, atractylenolide II, atractylenolide III, and pachymic acid. Separation was achieved using an ACQUITY UPLC BEHC_18_ column (2.1 mm × 100 mm, 1.7 **μ**m) with a gradient elution program consisting of acetonitrile and 0.1% phosphoric acid solution. The detection wavelengths were set at 203, 254, 222, and 267 nm. The method was validated for linearity, accuracy, precision, limit of detection, and limit of quantification. The validated method was successfully applied to the simultaneous quantification of ten active compounds from several finished batches of SJZT. This validated that UPLC method is expected to provide a new basis for the quality control of SJZT.

## 1. Introduction 

Traditional Chinese herbal formulation (TCMF) has been widely used in the clinic for its well-proven efficacy with few side effects. Sijunzi decoction (SJZT) is one of the most famous TCMFs consisting of four herbs:* Radix Ginseng, Poria cocos*, Rhizoma Atractylodis Macrocephalae, and Radix Glycyrrhizae. In China, SJZT has long been used for the treatment of gastrointestinal disorders such as chronic gastritis and gastric and duodenal ulcer, and it could effectively attenuate nausea, vomiting, and diarrhea [1]. Clinical studies show that SJZT could effectively restore the homeostasis of the digestive tract in patients [2]. More recently, SJZT has been shown to ameliorate the intestinal flora disturbance in rat models of spleen deficiency syndrome [3]. Moreover, emerging evidences are showing that SJZT and modified SJZT could play a good supporting role in suppressing tumors and confer a protective effect on gastrointestinal mucosa damage induced by chemotherapy [4].

Supporting these well-confirmed pharmacological efficacies, recent years have seen an increasing knowledge of the chemical components from SJZT. HPLC-MS was exploited to analyze the major components of SJZT, and eight ginsenosides (ginsenosides Rg_1_, Re, Rf, Ro, Rb_1_, Rc, Rb_2_, and Rd) and glycyrrhizic acid were identified through structural elucidation [1]. Recently, by employing the UPLC-Q-TOF-MS technique, 66 phytochemical compounds were detected in Sijunzi decoction formula and 58 of them including ginsenosides, flavonoids, triterpenoid, and coumarins were tentatively identified by comparing the accurate mass and fragment information with the correlative references data [5]. It should be noted that the constituents and contents of the main active components existing in SJZT may be influenced by harvest time, plant origin, and manufacturing procedures, which could significantly affect the pharmacological effects and necessitate the quality assessment of SJZT. Undoubtedly, it is not easy to simultaneously determine all the components existing in the formula. However, simultaneous analysis of the main active components may be one possible solution. Previous studies have quantitatively determined the main components existing in the four individual herbs of SJZT, namely,* Radix Ginseng *[6, 7],* Poria cocos *[8], Rhizoma Atractylodis Macrocephalae[9, 10], and* Glycyrrhiza uralensis *[11–13], by using HPLC or UPLC methods. However, a satisfactory quantitative method of the major active components in SJZT for quality control purposes is not available. Simultaneous analysis for the main active compounds in each herb of SJZT has been suggested as one possible solution.

The aim of this research was to develop a convenient, reliable, and sensitive analytical method to determine the quantity of major compounds in SJZT by using ultraperformance liquid chromatography (UPLC). Specifically, ginsenoside Rg_1_, Re, Rb_1_, liquiritin, liquiritigenin, glycyrrhizic acid, atractylenolide I, atractylenolide II, atractylenolide III, and pachymic acid were selected as the marker constituents for the relatively high contents in the individual herbs and their validated pharmacological effects, such as anti-inflammation, brain protection effects, antioxidation effect, and hypoglycemic effect [1, 5]. The potential application of this study could not only support a quality control of SJZT but also provide a theoretical basis for further in-depth research of SJZT in clinical research.

## 2. Experimental 

### 2.1. Reagents and Chemicals

**Figure 1:**
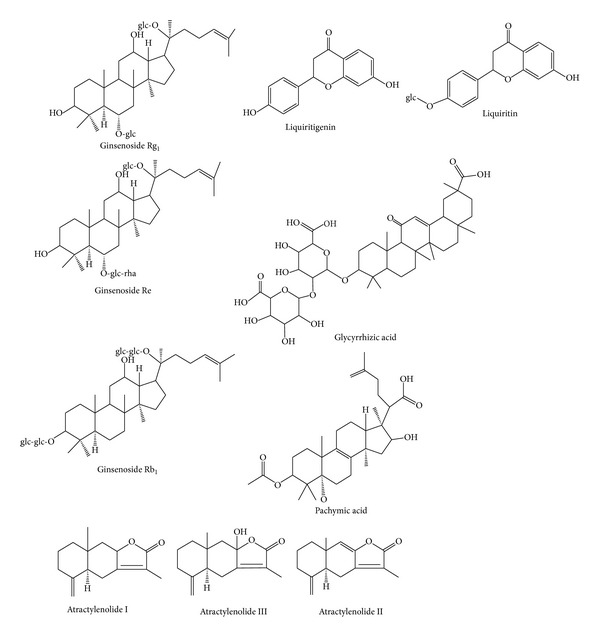
The chemical structure of ginsenoside Rg_1_, Re, Rb_1_, liquiritin, liquiritigenin, glycyrrhizic acid, atractylenolide I, atractylenolide II, atractylenolide III, and pachymic acid.

The four crude herbs,* Radix Ginseng*,* Poria cocos, *Rhizoma Atractylodis Macrocephalae, and* Glycyrrhizae uralensis*, were purchased from Nanjing Traffic Hospital (Nanjing, China). All samples were identified by one of the authors (Professor Wang Xiao-Long) as authentic herbal medicine. Ginsenoside Rg_1_, Re, and Rb_1_ were purchased from Jilin University (Changchun, China); liquiritin, liquiritigenin, and glycyrrhizic acid were purchased from Chinese Food and Drug Inspection Institute; Atractylenolide III, Atractylenolide I, Atractylenolide II, and pachymic acid were purchased from Sichuan Weikeqi Biological Co., Ltd (Chengdu, China). The ten compounds used in the analysis were of analytical grade and their purity was more than 98%. Their chemical structures are shown in Figure 1. Acetonitrile and methanol (HPLC grade) were purchased from Fisher Scientific (Waltham, MA, USA); phosphoric acid (analytical grade) was purchased from Nanjing Chemical Regents Company (Nanjing, China); water was purified by a Millipore Milli-Q system (Millipore, MA, USA); other reagents and chemicals were all obtained from various commercial sources and were of analytical grade.

### 2.2. Chromatographic Analysis

The analytes were separated on an ACQUITY UPLC H-class system with a PDA detector using 2.1 mm × 100 mm × 1.7 *μ*m ACQUITY BEH C18 column with a flow rate of 0.3 mL min^−1^. Separation was achieved using a gradient method. Acetonitrile (A) and 0.1% phosphoric acid (B) were selected and the gradient solvent system was as follows: 0–5 min, (A) 19%; 5–13 min, 19%–30%; 13–25 min, 30%–50%; 25–33 min, 50%–60%; 33–37 min, (A) 60%–68%; 37–39 min, (A) 68%–19%; and 30–40 min, (A) 19%. The injection volume of samples was 5 *μ*L and temperature of the column oven was maintained at 30°C. The UV wavelength was set at 203 nm (for ginsenoside Rg_1_, Re, and Rb_1_), 254 nm (for liquiritin, glycyrrhizic acid, and liquiritigenin), 222 nm (for atractylenolide I and III), 276 nm (for atractylenolide II), and 242 nm (for pachymic acid).

### 2.3. Preparation of SJZT Samples

According to the original composition of SJZT, the four constituting herbs including* Radix Ginseng *(100 g),* Poria cocos *(100 g), Rhizoma Atractylodis Macrocephalae(100 g), and* Glycyrrhizae uralensis* (50 g) were crushed into small pieces and then mixed and decocted twice in 3500 mL water for 1 h in a glass flask. The decoction was filtered through 8 layers of gauze; the filtrate was concentrated in vacuum at 60°C at a final concentration of 2 g/mL.

Ethanol was added to an aliquot of 5 mL SJZT overnight in order to remove the polysaccharides. The supernatant was transferred into a test tube and evaporated to dryness with vacuum at room temperature. Finally, the residue was reconstituted in 5 mL methanol by vortex mixing for 5 min and centrifuged at 16,000 rpm for 10 minutes. 5 *μ*L supernatant was injected into chromatographic systems for analysis.

### 2.4. Preparation of Standard Solutions

The standard stock solutions of ginsenoside Rg_1_ (300.00 *μ*g/mL), ginsenoside Re (302.10 *μ*g/mL), ginsenoside Rb_1_ (300.00 *μ*g/mL), liquiritin (90.30 *μ*g/mL), liquiritigenin (87.00 *μ*g/mL), glycyrrhizic acid (110.00 *μ*g/mL), atractylenolide III (59.00 *μ*g/mL), atractylenolide I (71.40 *μ*g/mL), atractylenolide II (50.60 *μ*g/mL), and pachymic acid (200.00 *μ*g/mL) were prepared in methanol. These solutions were stored at 4°C and were stable for at least 1 month.

### 2.5. Preparation of Negative Control Samples of SJZT

The negative control samples of SJZT were prepared by deriving one herb from the prescriptions. The herbs were accurately weighed according to the prescription of SJZT and prepared with the same procedure as for the sample preparation.

### 2.6. Method Validation

Specificity, linearity, limit of detection (LOD), limit of quantification (LOQ), precision (repeatability and intra- and interassay), and accuracy (recovery) of this UPLC method were evaluated in accordance with International Conference on Harmonization (ICH) [14].

The standard calibration curve for the linearity assay was prepared with seven different concentrations of diluted standard solutions (ginsenoside Rg_1_, ginsenoside Re, ginsenoside Rb_1_, liquiritin, glycyrrhizic acid, liquiritigenin, atractylenolide I, atractylenolide II, atractylenolide III, and pachymic acid). The lower limit of quantification (LLOQ) was determined as the lowest concentration point of the standard curve and the signal-to-noise ratio was higher than 10. The lower limit of detection (LLOD) was defined as the amount that could be detected with a signal-to-noise ratio of 3.

The precision of the analytical method was evaluated by intrabatch and interbatch variability. Three different concentrations of standards (low, medium, and high) were prepared. The quantity of each component was determined by the respective calibration curve. RSD was used to measure precision. The interbatch reproducibility test was carried out on three different batches.

Recovery studies were carried out by spiking three concentrations of mixed standards at low (50% of the known amounts), medium (100% of the known amounts), and high (200% of the known amounts) in the 5 mL of SJZT. Then, the spiked samples were then extracted, processed, and quantified in accordance with the methods mentioned above.

## 3. Results and Discussion

### 3.1. Optimization of the UPLC Conditions

Optimization of the separation conditions for HPLC analysis was performed including the mobile phase composition, gradient elution program, and wavelength. To obtain chromatograms with better resolution of adjacent peaks within shorter time, the chromatographic conditions were optimized. Methanol and acetonitrile were compared in the experiment. The result showed that acetonitrile was much better as it could result in a better resolution and shorter time for analysis. In addition, water and 0.1% phosphoric acid/water were investigated and the result showed that 0.1% phosphoric acid/water was better than water. As a result ACQUITY UPLC BEH C_18_ column (2.1 mm × 100 mm, 1.7 *μ*m) with acetonitrile and 0.1% phosphoric acid/water was selected as the preferred chromatographic conditions.

Moreover, different gradient profiles were also optimized. Actually, we tried to simplify the gradient elution system and shorten the analysis time, but peaks for atractylenolide III and atractylenolide I have not been completely separated except for the current condition to in the gradient program mentioned above.

In this experiment, the specificity of UV absorption was also investigated, using the present chromatographic conditions and comparing a SJZT sample with a standard mixture. The UV absorbance and the best UV detection wavelength of each compound in SJZT were confirmed as follows: ginsenoside Rg_1_, Re, and Rb_1_ (203 nm), liquiritin, glycyrrhizic acid, and liquiritigenin (254 nm), atractylenolide I and atractylenolide III (222 nm), atractylenolide II (276 nm), and pachymic acid (242 nm).

### 3.2. Validation of the UPLC Method

The validation study allowed the evaluation of the method for its suitability for routine analysis.

#### 3.2.1. Specificity

**Figure 2:**
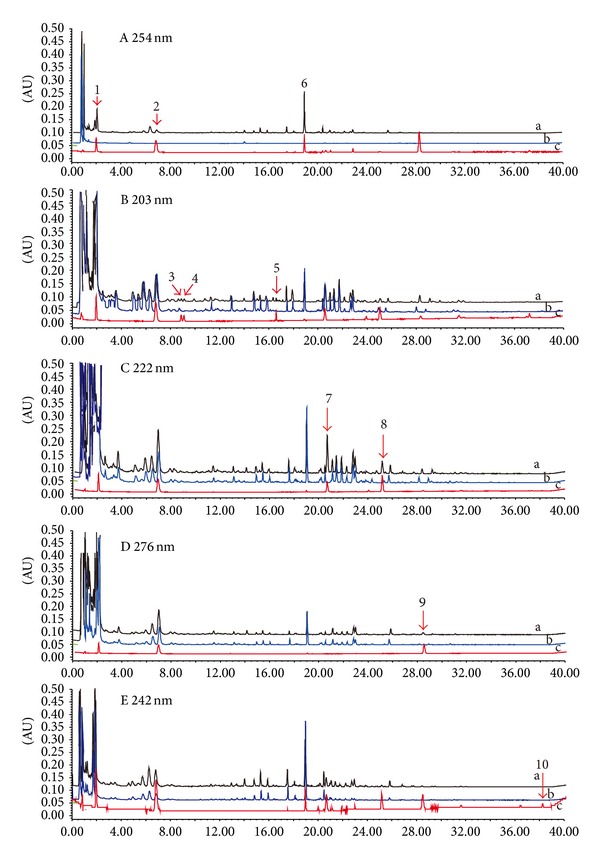
Ultraperformance liquid chromatography (UPLC) chromatograms at different wavelengths of standard mixture (a), SJZT extraction (b), and negative control samples (c). Peaks: (1) liquiritin, (2) liquiritigenin, (3) ginsenoside Rg_1_, (4) ginsenoside Re, (5) ginsenoside Rb_1_, (6) glycyrrhizic acid, (7) atractylenolide III, (8) atractylenolide I, (9) atractylenolide II, and (10) pachymic acid.

Representative chromatograms of the standard solution, sample solution, and negative control samples at different UV wavelength were shown in Figure 2. The chromatographic peaks were identified by comparing their retention time with that of each reference compound. In addition, chromatograms of the negative control samples further confirmed the specificity of this method.

#### 3.2.2. Calibration Curves, LODs, and LOQs. 

The linearity of the developed method was assessed using seven different concentrations each of ginsenoside Rg_1_, ginsenoside Re, ginsenoside Rb_1_, liquiritin, glycyrrhizic acid, liquiritigenin, atractylenolide I, atractylenolide II, atractylenolide III, and pachymic acid and the observed concentrated ranges were as follows: 4.68∼300.00, 4.72∼302.10, 4.68∼300.00, 1.41∼90.30, 1.72∼110.00, 1.36∼87.00, 1.12∼71.40, 0.79∼50.60, 0.92∼59.00, and 3.13∼200.00 *μ*g/mL, respectively. The correlation coefficient (*R* ^2^) of all biomarkers has good linearity of over > 0.9996 in the aforementioned ranges. The LODs and LOQs of the ten analytes were 0.01∼0.07 and 0.03∼0.23 *μ*g/mL, respectively (Table 1).

#### 3.2.3. Precision, Repeatability, and Stability

Intraday and interday variations were chosen to determine the precision of the developed assay. The analyzed data showed that relative standard deviation (RSD) of intra- and interday was in the range of 0.21–1.18% and 0.20–1.10% (*n* = 6), the RSD of the SJZT repeatability, and stability test was in the range of 0.20–0.89% and 0.57%–1.04%, respectively (Table 2). None of the precision, repeatability, and stability data exceeded 5%, except atractylenolide II and pachymic acid (the content of those two components were lower than the LODs).

#### 3.2.4. Accuracy

The accuracy of the method was assessed by a recovery assay. The spiked samples were then extracted, processed, and quantified in accordance with the methods mentioned above. The measured data showed that the recovery of the investigated components ranged from 95.07% to 102.67%, and their RSD values were all less than 3.0% (Table 3). Recovery data represented the accuracy of the method and is sufficient for usual analysis

#### 3.2.5. Applications

The established analytical method was subsequently applied for the simultaneous determination of the ten markers in 3 batches of SJZT. The results are presented in Table 4. The results showed that there are remarkable differences among the contents of the ten components in SJZT from the same or different batches.

## 4. Conclusion

In this study, a UPLC method for the simultaneous determination of ten active ingredients in SJZT has been developed and the results showed that it could be used for the quality control of the SJZT. Thus, this validated that UPLC method could be expected to provide a new basis for the quality control of SJZT.

## Figures and Tables

**Table 1:** The linear regression data, LODs, and LOQs of ten compounds.

Components	Regression equations	*R* ^2^	Linear range (*μ*g/mL)	LODs (*μ*g/mL)	LOQs (*μ*g/mL)
Ginsenoside Rg_1_	*y* = 2123.1*x* + 1493.3	1	4.68–300.00	0.07	0.23
Ginsenoside Re	*y* = 1701.3*x* + 2520.7	0.9998	4.72–302.10	0.07	0.23
Ginsenoside Rb_1_	*y* = 1465*x* + 2077.4	0.9998	4.68–300.00	0.06	0.20
Liquiritin	*y* = 4334.7*x* + 1378.8	0.9998	1.41–90.30	0.02	0.07
Liquiritigenin	*y* = 7817.1*x* + 3469.8	0.9998	1.36–87.00	0.02	0.07
Glycyrrhizic acid	*y* = 3181.9*x* + 1888	0.9998	1.72–110.00	0.03	0.10
Atractylenolide III	*y* = 34419*x* + 6432.6	0.9999	0.92–59.00	0.01	0.03
Atractylenolide I	*y* = 18610*x* + 8178.9	0.9998	1.12–71.40	0.02	0.07
Atractylenolide II	*y* = 40016*x* + 9753.9	0.9999	0.79–50.60	0.01	0.03
Pachymic acid	*y* = 2060.1*x* + 3937.9	0.9996	3.13–200.00	0.05	0.16

*y* = *Ax* + *B*; *y* is peak area; *x* is concentration of the analytes (*μ*g/mL); *r* is the correlation coefficient of the equation.

**Table 2:** Precision, repeatability, and stability of ten compounds in SJZT (*n* = 6).

Analytes		Precision		Repeatability RSD (%)	Stability RSD (%)
	Levels (ug/mL)	Intraday RSD (%)	Interday RSD (%)		
Ginsenoside Rg_1_	7.03	1.05%	0.29%	0.98%	1.01%
	37.50	0.30%	0.20%		
	240.00	0.85%	1.04%		

Ginsenoside Re	7.08	0.60%	0.22%	0.87%	1.04%
	37.76	0.36%	0.42%		
	241.68	0.81%	0.93%		

Ginsenoside Rb_1_	7.03	0.28%	0.22%	1.20%	0.92%
	37.50	0.17%	0.16%		
	240.00	1.15%	0.98%		

Liquiritin	2.12	0.33%	0.70%	0.48%	0.57%
	11.29	0.54%	0.35%		
	72.24	1.18%	1.03%		

Liquiritigenin	2.04	0.83%	0.70%	0.27%	0.92%
	10.88	0.39%	0.49%		
	69.60	0.39%	0.31%		

Glycyrrhizic acid	2.58	0.91%	1.04%	0.28%	0.73%
	13.75	0.21%	1.13%		
	88.00	0.71%	0.72%		

Atractylenolide III	1.38	1.08%	0.46%	0.63%	0.84%
	7.38	0.96%	0.50%		
	47.20	0.93%	0.83%		

Atractylenolide I	1.67	1.03%	0.58%	0.81%	0.92%
	8.93	1.07%	0.97%		
	57.12	0.87%	0.72%		

Atractylenolide II	1.19	1.03%	0.64%		
	6.33	0.63%	0.62%		
	40.48	0.97%	0.72%		

Pachymic acid	4.69	0.52%	0.48%		
	25.00	0.97%	1.10%		
	160.00	0.61%	0.37%		

RSD: relative standard deviation.

**Table 3:** Recovery of eight components in Sijunzi (*n* = 3).

Components	Contents (ug/mL)	Quantity added (ug/mL)	Theoretical amount (ug/mL)	Recorded amount (ug/mL)	Recovery (%)	RSD (%)
Ginsenoside Rg_1_	64.83	37.5	102.33	102.42	100.25	0.65
	64.83	75	139.83	139.44	99.48	0.78
	64.83	150	214.83	210.05	96.81	0.54

Ginsenoside Rb_1_	48.17	37.5	85.67	85.24	98.85	0.67
	48.17	75	123.17	119.96	95.72	0.82
	48.17	150	198.17	191.81	95.76	0.87

Ginsenoside Re	61.63	18.88	80.51	80.95	102.33	0.53
	61.63	37.76	99.39	99.73	100.9	0.20
	61.63	75.53	137.16	137.13	99.96	0.26

Liquiritin	57.39	22.58	79.97	78.98	95.62	0.76
	57.39	45.15	102.54	100.78	96.1	0.87
	57.39	90.3	147.69	143.72	95.6	0.84

Liquiritigenin	47.48	21.75	69.23	69.27	100.18	0.25
	47.48	43.5	90.98	91.67	101.59	0.79
	47.48	87	134.48	136.8	102.67	0.89

Glycyrrhizic acid	111.18	55.00	166.18	164.12	96.25	0.62
	111.18	110.00	221.18	217.71	96.84	0.63
	111.18	220.00	331.18	330.52	99.7	0.36

Atractylenolide III	42.08	29.50	71.58	72.45	102.95	0.83
	42.08	59.00	101.08	100.81	99.54	0.30
	42.08	118.00	160.08	160.29	100.18	0.24

Atractylenolide I	10.50	8.93	19.43	19.37	99.33	0.21
	10.50	17.85	28.35	28.47	100.67	0.20
	10.50	37.50	48.00	46.15	95.07	0.62

Recovery (%) = (recorded amount − original amount)/spiked amount ∗ 100%.

**Table 4:** Contents of eight components in three batches of SJZT.

Sample	Source	Contents (mg/g)							
		Ginsenoside Rg_1_	Ginsenoside Re	Ginsenoside Rb_1_	Liquiritin	Liquiritigenin	Glycyrrhizic acid	Atractylenolide III	Atractylenolide I
1	Batch 1	0.254	0.115	0.184	0.659	0.078	0.258	0.020	0.013
2	Batch 2	0.445	0.185	0.261	0.858	0.100	0.345	0.043	0.017
3	Batch 3	0.209	0.094	0.116	0.600	0.067	0.572	0.039	0.011
